# Accuracy of a Smartphone-Based Artificial Intelligence Application for Classification of Melanomas, Melanocytic Nevi, and Seborrheic Keratoses

**DOI:** 10.3390/diagnostics13132139

**Published:** 2023-06-21

**Authors:** Jokubas Liutkus, Arturas Kriukas, Dominyka Stragyte, Erikas Mazeika, Vidas Raudonis, Wolfgang Galetzka, Andreas Stang, Skaidra Valiukeviciene

**Affiliations:** 1 Department of Skin and Venereal Diseases, Lithuanian University of Health Sciences, 44307 Kaunas, Lithuania; 2 Department of Skin and Venereal Diseases, Hospital of Lithuanian University of Health Sciences Kauno Klinikos, 50161 Kaunas, Lithuania; 3 Artificial Intelligence Center, Kaunas University of Technology, 51423 Kaunas, Lithuania; 4 Institute of Medical Informatics, Biometrics and Epidemiology, University Hospital Essen, 45130 Essen, Germany

**Keywords:** melanoma, nevus, seborrheic keratosis, dermatoscopy, artificial intelligence, smartphone

## Abstract

Current artificial intelligence algorithms can classify melanomas at a level equivalent to that of experienced dermatologists. The objective of this study was to assess the accuracy of a smartphone-based “You Only Look Once” neural network model for the classification of melanomas, melanocytic nevi, and seborrheic keratoses. The algorithm was trained using 59,090 dermatoscopic images. Testing was performed on histologically confirmed lesions: 32 melanomas, 35 melanocytic nevi, and 33 seborrheic keratoses. The results of the algorithm’s decisions were compared with those of two skilled dermatologists and five beginners in dermatoscopy. The algorithm’s sensitivity and specificity for melanomas were 0.88 (0.71–0.96) and 0.87 (0.76–0.94), respectively. The algorithm surpassed the beginner dermatologists, who achieved a sensitivity of 0.83 (0.77–0.87). For melanocytic nevi, the algorithm outclassed each group of dermatologists, attaining a sensitivity of 0.77 (0.60–0.90). The algorithm’s sensitivity for seborrheic keratoses was 0.52 (0.34–0.69). The smartphone-based “You Only Look Once” neural network model achieved a high sensitivity and specificity in the classification of melanomas and melanocytic nevi with an accuracy similar to that of skilled dermatologists. However, a bigger dataset is required in order to increase the algorithm’s sensitivity for seborrheic keratoses.

## 1. Introduction

Melanoma continues to be a burden on healthcare systems, and it has a projected 50% increase in incidence and a 68% increase in mortality by 2040 [1]. Early and accurate diagnoses are crucial for improving patient outcomes and reducing mortality rates associated with this aggressive malignancy. Melanoma diagnostics are based on dermatoscopy before a histopathological confirmation [2]. Thus, it is important to accurately distinguish melanomas from benign lesions, such as melanocytic nevi and seborrheic keratoses. Seborrheic keratoses are the most common type of skin lesion encountered among the elderly [3]. However, the adoption of dermatoscopy in primary care remains low. Only 8.3% of family physicians apply dermatoscopy in their daily practice [4], although its use increases the sensitivity of the diagnosis of melanomas from 57.8% to 75.9% [5]. The primary barriers to a wider adoption in primary care are a lack of training and the perceived difficulty of dermatoscopy [6].

The advancement of deep learning in computer vision has led to artificial intelligence (AI) algorithms that have shown a superior effectiveness to that of dermatoscopy [7] performed by both beginner and expert dermatologists [8]. These algorithms are generally based on deep neural networks (DNNs), which automatically extract discriminating image features during supervised learning from prelabeled data and compute diagnostic probabilities. DNN-based algorithms generally perform better than other machine learning algorithms, which are based on manually selected image features [9]. The process of feature selection and labeling for machine learning algorithms is often labor-intensive and may not capture all the relevant information. In contrast, DNNs are capable of automatically learning to identify and extract relevant features from raw input images during the training process. However, the primary drawback of DNNs is the requirement for large, labeled training datasets of skin tumors, which need to be verified through histopathology [10].

In 2016, a particular type of DNN termed “You Only Look Once” (YOLO) was introduced. Notably, this subclass of DNNs has demonstrated a superior efficacy in object detection tasks, owing to its unique capacity to concurrently undertake both localization and classification within a singular processing pass [11]. This allows YOLO networks to process the entire image during both the training and testing phases, incorporating contextual information about the object classes and their visual characteristics into its decision-making process. This attribute is particularly beneficial in medical imaging, where subtle contextual cues can often be crucial for accurate diagnoses. YOLO-based neural networks have been assessed for the detection of vitiligo [12] and the segmentation of skin lesions [13], including melanomas [14], and they have outclassed other DNN approaches. However, the accuracy of skin lesion classification when using a YOLO-based neural network has not been evaluated to date, nor has it been integrated into a smartphone health application that is suitable for dermatoscopy.

Previous studies comparing other types of DNNs with dermatologists have frequently used a binary lesion (malignant vs. benign) classification using convolutional neural networks (CNNs) on dermatoscopic images [15,16,17,18]. In an early study, Brinker et al. [15] assessed the performance of a CNN trained on open-source images, which outperformed 87% of the 157 participating dermatologists. Marchetti et al. [16,17] compared the results of two international DNN lesion classification tournaments, each involving 25 and 23 teams, with the classification performance of specialists. The top algorithms in each study achieved higher accuracies than the dermatologists. In a recent study, Winkler et al. [18] evaluated whether 22 dermatologists could benefit from cooperation with a market-approved CNN in classifying prospectively collected images of melanocytic lesions. The accuracy significantly improved when the dermatologists applied CNN results into decision making.

The benefit of CNNs in dermatological practice seems evident; however, in primary care, family physicians and other care providers also encounter multiple types of skin lesions. A tool that can correctly classify each skin tumor would be even more useful than binary classification into malignant and benign lesions. Therefore, other researchers have focused on dermatoscopic multiclass classification approaches for different skin tumors, which allow the algorithm to display results for multiple disease categories [19,20]. In one of the first studies, Esteva et al. used a CNN that achieved accuracies similar to those of trained dermatologists [19]. The most recent study, describing an international CNN lesion classification tournament, compared the performance of 129 different algorithms to that of 18 dermatologists [20]. The algorithms performed better than the experts at classifying melanomas, melanocytic nevi, and seborrheic keratoses.

The majority of the DNNs employed in the aforementioned studies ran on specialized research software and hardware that is difficult and cumbersome to use in primary care. However, several studies [21,22,23,24,25,26,27,28] employed smartphone-enabled approaches for classifying skin tumors using CNNs, as mobile applications on portable devices are much easier to use for both patients and physicians. Haenssle et al. [21,22,23,24,25] used the patented and approved Moleanalyzer Pro^®^ (FotoFinder Systems GmbH, Bad Birnbach, Germany) algorithm. It determines a skin lesion’s “malignancy score”, which ranges from 0 to 1 and has an arbitrary cutoff of >0.5 for classifying a skin lesion as malignant, with 95.0% sensitivity and 76.7% specificity [22]. However, Moleanalyzer Pro^®^ is only available on FotoFinder© dermatoscopic devices, and thus requires a commitment to a hardware–software ecosystem. Similarly, Veronese et al. evaluated a CNN paired with a Nurugo© Derma (UNIONCOMMUNITY Co. Ltd., Seoul, Republic of Korea) smartphone dermatoscopic device, which achieved 84.0% sensitivity and 81.6% specificity for melanomas [26]. Other smartphone-application-based CNN studies were performed for the analysis of non-dermatoscopic clinical images [27,28]. Therefore, there is an absence of studies showing the accuracy of a multiclass classification algorithm that can be used on any smartphone device without the need for specific dermatoscopic hardware.

The aim of the current study was to evaluate the accuracy of a YOLO neural network model (NNM) that is fully embedded into a smartphone application for the multiclass dermatoscopic classification of melanomas, melanocytic nevi, and seborrheic keratoses. In addition to evaluating the NNM’s performance against histopathologically confirmed diagnoses, this study compared its accuracy with that of skilled dermatologists and beginner raters.

## 2. Materials and Methods

### 2.1. Ethical Approval

This study was approved by the local ethics committee, approval number P2-BE-2-25/2009, and was carried out in accordance with the Declaration of Helsinki.

### 2.2. Skin Lesion Classification Model and Dataset

**Figure 1:**
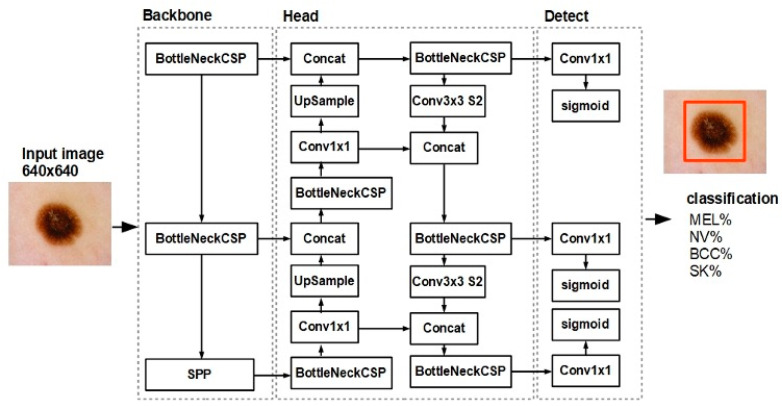
Deep network structure of the skin lesion classification model. BottleNeckCSP—cross-stage partial convolutional network; SPP—spatial pyramid pooling; Concat—concatenation neural layer; UpSample—upsampling neural layer; Conv—convolutional layer; Sigmoid—sigmoidal transfer function; MEL—melanoma; NV—melanocytic nevus; SK—seborrheic keratosis; and BCC—basal cell carcinoma.

The YOLO model (NNM) used a one-stage model, in which object detection (localization) and classification were performed in a dense sampling layer (Figure 1). The proposed model was designed to automatically extract features from input images; then, from these features, the prediction layers determined the location and class of each skin lesion. The structure of the applied model consisted of three main parts—the backbone, head, and detection elements. The backbone was used to extract discriminative features from the input image. It was mainly based on a BottleNeckCSP convolutional neural network that aggregated and formed image features at different granularities. BottleNeckCSP models are based on a DenseNet network [29], which is designed to connect neural layers with the goals of avoiding the vanishing gradient problem, bolstering feature propagation, and reducing the number of network parameters. The head component of the YOLO model extracted fusion features and passed them forward to the classification and detection parts. The head element consisted of a series of convolutional layers, such as Conv1 × 1 (convolution using a 1 × 1 filter), Conv3 × 3 (convolution using a 3 × 3 filter), a merging concatenation neural layer, an upscaling layer (UpSample), and the previously described BottleNeckCSP layer. Detection was achieved in the last part of the model structure. The detection analyzed features by using a fully connected layer (Conv1 × 1) and a sigmoidal transfer function; finally, it had location boxes with prediction values as its output. The detection employed the total loss function of the bounding box and non-maximum suppression [30].

Binary cross-entropy with the logits loss function was used as a metric to evaluate how well the proposed skin lesion detection and classification model was trained. When the predictions of the YOLO model are closer to the true annotated values, the selected metric (loss function) will be at a minimum. If the predictions do not correspond with actual values, the loss function value will reach the maximum. The training parameters of the YOLO model are updated based on the values of the loss function. The binary cross-entropy loss function was used to measure the dissimilarity between the predicted probability distribution and the true labels in the training dataset. The predicted probabilities were compared to the actual class values by calculating the score that penalized the probabilities based on the distance from the expected value. The binary cross-entropy value (*Loss*) was calculated using the formula given below:

(1) $$Loss=\frac{1}{N}\sum_{i=1}^{N}{-[y}_{i}\cdot \log(p(y_{i}))+(1-y_{i})\cdot \log\left(1-p(y_{i}))\right]$$

where *N* is the number of samples in the training dataset (or output size when used in training with data batches), *y* is a class label, and *p*(*y*) is the model output or prediction that the given input corresponds to the actual label.

The given loss function was adapted for the multiclass classification problem and the loss value was calculated using the following formula:

(2) $$mLoss=-\frac{1}{N}\sum_{i}^{N}\sum_{j}^{M}y_{ij}\cdot \log\left(p(y_{ij})\right)$$

where *N* is the number of samples, *M* is the number of classes, *y* is a class label, and *p*(*y*) is the predicted probability of the YOLO model.

The average difference between the actual and predicted probability distributions for all classes was calculated using Equation (2).

The proposed YOLO detection and classification model was trained using the gradient descent optimization algorithm (SGD). The SGD is one of the most common ways to optimize deep neural networks, and it is used as a black-box optimizer. Gradient descent is a way to minimize an objective function, described using the neural network model, by updating the parameters in the opposite direction of the gradient.

The training hardware environment consisted of a single NVIDIA GeForce RTX3080 Ti (Santa Clara, CA, USA) graphical card with 12 GB of memory and an Intel i9 (Santa Clara, CA, USA) CPU processor. The NNM was implemented using a torch 1.81 + cu101 CUDA python library. Hyperparameters, such as a number of epochs of 300, a batch size ranging from 4 to 16, an input image of 640 × 640 pixels, and initial and final learning rates of *l*_0_ = 0.01 and *l*_f_ = 0.2, were used in training.

From a computational point of view, the feasibility of the results depended on the implementation. It involved a complex algorithm, such as the largest YOLO structure, and different operating systems. In addition, the processing of color images of the lesions required substantial computational resources and time (which depended on the processor’s CPU or GPU) to produce accurate results. The computation complexity of the results was evaluated using several important factors, such as the number of inquiries (number of images that should be processed) and the computational resources available. The dataset was very large; thus, the use of parallel processing and distributed computing were required to achieve feasible results within a reasonable timeframe. We tested several machines: (1) a desktop computer with a 12th Gen Intel CPU i9, 64 GB of RAM, and NVIDIA RTX4090 with 24 GB of RAM; (2) a laptop computer with an Intel i5 1.60 GHz CPU and 16 GB of RAM (no dedicated GPU processor); (3) an Apple iPhone X (Cupertino, CA, USA); and (4) a Samsung Galaxy A25 (Seoul, Republic of Korea). The highest image-processing response rates (on average, less than 0.15 s) were acquired using the desktop computer. Approximately less than 0.7 s were needed to process the images using either smartphone. The lowest processing rate was 1.5 s, achieved using a laptop computer.

Ultimately, the results were presented in the form of a data vector, which held information about the detected object’s class, score (confidence level ranging from 0.0 to 1.0), location (*x, y*), and size (width, height). The YOLO model within SmartVisSolution© (Kaunas, Lithuania) is available as a closed beta on personal computers and the Apple Inc. iOS (Cupertino, CA, USA) and Open Handset Alliance Android (Mountain View, CA, USA) smartphone operating systems. SmartVisSolution© was developed by a consulting and software development company named “Dts solutions” (Kaunas, Lithuania) and the Lithuanian University of Health Sciences.

The NNM training dataset was formed by using dermatoscopic images from the International Skin Imaging Collaboration (ISIC) archive (*n* = 58,457) [31,32,33], which consisted of 5106 melanomas, 18,068 melanocytic nevi, 1525 seborrheic keratoses, 3323 basal cell carcinomas (BCCs), 628 squamous cell carcinomas, and 29,807 uncategorized benign tumors that were captured by using various dermatoscopic devices. The ISIC archive’s MSK [34,35] and UDA [36] sub-databases were excluded from training. The dataset was subsequently expanded with dermatoscopic images (*n* = 633; 183 melanomas, 68 BCCs, 353 melanocytic nevi, and 29 seborrheic keratoses), which were collected with a FotoFinder © dermatoscopic device. The dermatoscopic images were retrospectively gathered from 2010 to 2020. All 251 of the dermatoscopic images of melanomas and BCCs were verified through histopathology. The remaining skin lesions were confirmed as benign by the expert opinion of two experienced dermatologists. Image augmentation techniques, such as image rotation, changes in illumination, and noise correction, were used to increase the size and diversity of the training data.

### 2.3. Test Dataset

**Figure 2:**
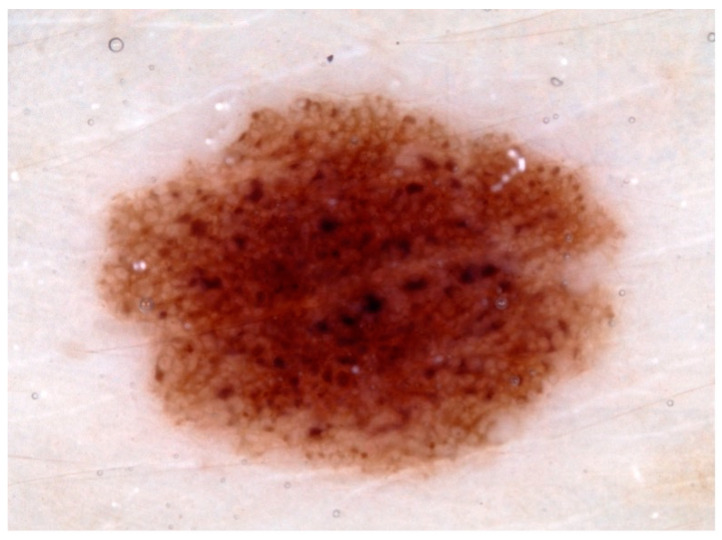
Example of a histologically confirmed melanocytic nevus in a dermatoscopic image from the test dataset (10× magnification, 1 scale bar = 1 mm).

The testing of the NNM was performed on 100 dermatoscopic images (Figure 2) of histologically confirmed melanomas (*n* = 32), melanocytic nevi (*n* = 35), and seborrheic keratoses (*n* = 33). The results of the NNM classification were compared with a histologically confirmed diagnosis, and with a blinded evaluation by two dermatologists who were skilled in dermatoscopy and five beginners in dermatoscopy. The dermatoscopic images were randomly selected from the HAM10000 [31], MSK-1, MSK-2, MSK-3, MSK-4, MSK-5 [34,35], and UDA2 [36] databases (Table 1). There was no overlap between the classification model’s training and testing datasets. To avoid image duplication within the ISIC datasets, the MSK and UDA databases were exclusively utilized for testing and were not included in the training dataset. For the HAM10000 dataset, 38 images were randomly selected from the HAM10000 dataset and excluded prior to training.

**Figure 3:**
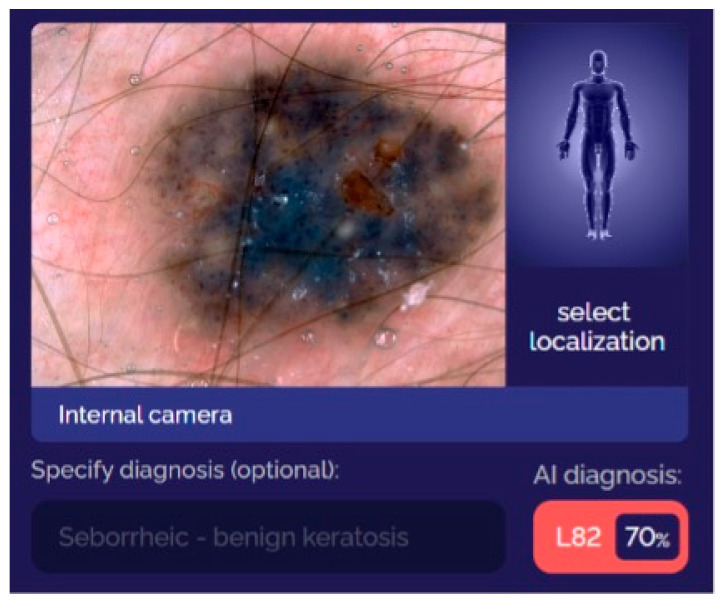
Demonstration of the smartphone application in use. A dermatoscopic image of a seborrheic keratosis was uploaded to the SmartVisSolution© application for classification. The outcome value was displayed by using the ICD-10 classification system. L82—seborrheic keratosis with a prognostic value of 70%.

The chosen dermatoscopic images were uploaded to the smartphone application and were cropped by using automatic selection of the skin tumor. The smartphone application resized each image to a resolution of 640 × 640 and output the classification probabilities and locations of four skin lesion classes—melanomas, melanocytic nevi, seborrheic keratoses, and BCCs (Figure 3).

The performance of the raters was evaluated by using a multiclass classification task. Each participant received a dermatoscopic image from the test dataset in a randomized order and was asked to assign one of the three diagnoses (melanoma, melanocytic nevus, or seborrheic keratosis) for all 100 images. The raters were also required to indicate the number of months of their experience in dermatoscopy for their placement in the “skilled” (>2 years of experience in dermatoscopy) or “beginner” (≤2 years of experience) groups.

### 2.4. Statistical Analysis

The outcome of interest was the sensitivity and specificity of the NNM and raters for the classification of melanomas, melanocytic nevi, and seborrheic keratoses. In addition, we computed the NNM’s area under the curve (AUC) of the receiver operating characteristic (ROC) curve. The raters’ sensitivity and specificity were analyzed within the “skilled” and “beginner” groups, in addition to an analysis of the pooled data presented as “all raters”. The chance-corrected inter-rater agreement was estimated with the Fleiss kappa. Performance was assessed by using the “one-vs-all” multiclass classification approach with absolute probability values.

To measure the accuracy of the NNM, we used a cutoff for the predicted probability such that the specificity of the model was equal to the mean specificity of the raters for the particular lesion (melanoma, melanocytic nevus, or seborrheic keratosis).

Point estimates are presented with a 95% confidence interval. The statistical analysis was carried out by using R, version 4.1.1 (R Foundation for Statistical Computing©, Vienna, Austria).

## 3. Results

Of the three skin lesion classes, the NNM achieved the highest sensitivity of 0.88 (0.71–0.96) when classifying melanomas, with a specificity of 0.87 (0.76–0.94) (Table 2). When assessing melanocytic nevi, the NNM achieved a sensitivity of 0.77 (0.60–0.90) and a specificity of 0.91 (0.81–0.97). The NNM’s sensitivity for seborrheic keratoses was 0.52 (0.34–0.69), with a high specificity of 0.93 (0.83–0.98). The differences in the sensitivity and specificity between the NNM and the raters are presented in Table 3. A negative value indicated that human raters outperformed the NNM. The biggest difference, favoring the NNM, was in the sensitivity of melanoma classification (0.11 (−0.08, 0.26)), while the lowest difference, favoring human raters, was in the sensitivity of seborrheic keratoses (−0.38 (−0.58, −0.02)). The sensitivities and specificities of each rater participating in the study are shown in Table 4.

**Figure 4:**
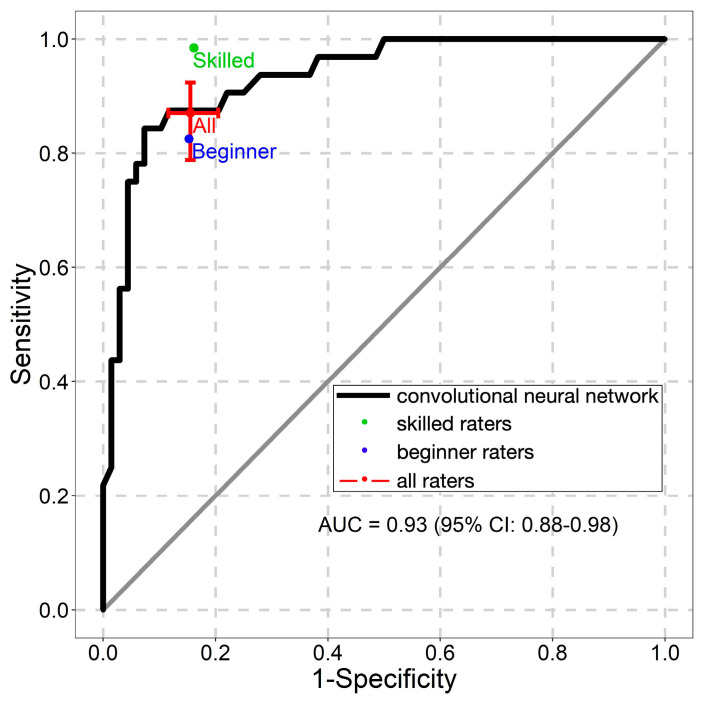
Receiver operating characteristic curve for the classification of melanomas. AUC—the area under the curve.

**Figure 5:**
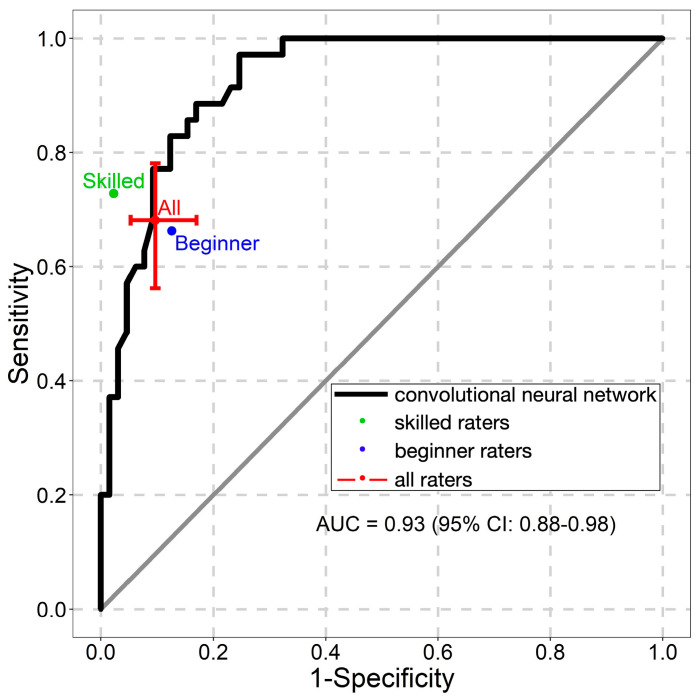
Receiver operating characteristic curve for the classification of melanocytic nevi. AUC—the area under the curve.

**Figure 6:**
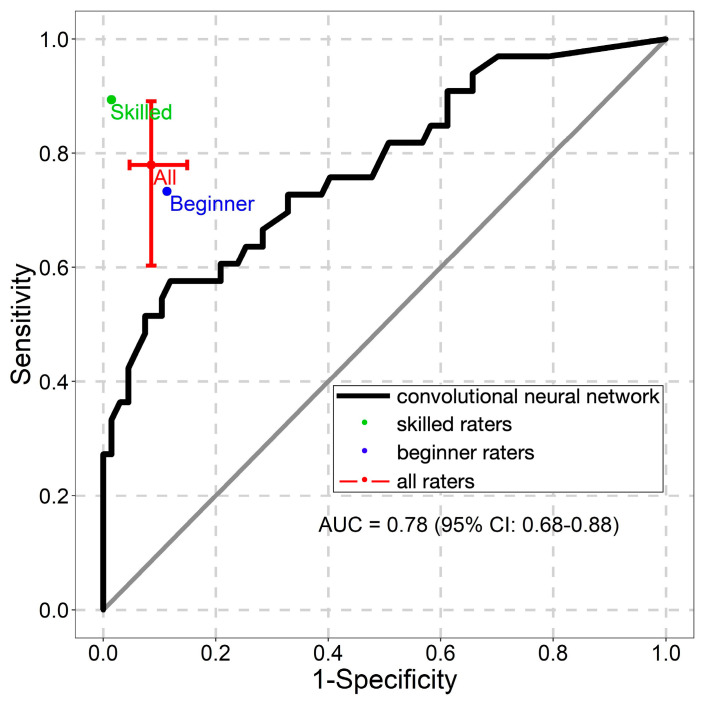
Receiver operating characteristic curve for the classification of seborrheic keratoses. AUC—the area under the curve.

The NNM’s ROC AUC values are presented in Figure 4, Figure 5 and Figure 6. The NNM classified melanomas and melanocytic nevi better than the beginner raters did, although its classification performance for seborrheic keratoses was lower than that of both groups of raters.

The inter-observer agreement using the Fleiss kappa values among different rater groups and skin lesions is shown in Table 5. The highest chance-corrected agreement was found among skilled raters for all three skin lesions. We used the most common division of kappa agreement values according to Landis and Koch [37]. The highest kappa value, achieving substantial rater agreement, was 0.79 (0.59–0.98) for the classification of seborrheic keratoses, and the lowest was in the beginner subgroup for melanocytic nevi (0.43 (0.37–0.50)), representing a moderate rater agreement. For all diagnoses combined, the raters achieved moderate agreement—a kappa value of 0.53 (0.50–0.56).

## 4. Discussion

Our study showed that the smartphone-based NNM achieved a high sensitivity and specificity in the classification of melanomas and melanocytic nevi, with a similar accuracy to that of skilled dermatologists. Conversely, the sensitivity was low for seborrheic keratoses—there was a smaller amount of labeled training data available. Additionally, the automatic selection of the skin tumor region by the NNM could have cropped important dermatoscopic features that were found at the seborrheic keratoses’ edges, including sharp demarcations or milia-like cysts [38]. In our analysis, the skilled dermatologists consistently outperformed the beginners in terms of accuracy.

The strengths of our study were the histopathological confirmation of all malignant skin lesions included in the test dataset and the use of multiclass classification, rather than binary classification into malignant and benign skin lesions, as in other studies [21,22,23,24,25]. In addition, we assessed raters of various skill levels; thus, we were able to provide multi-rater statistics and compare the NNM’s results to those of both beginners and experienced specialists. Furthermore, this is the first study describing the accuracy of the multiclass classification of skin lesions using a YOLO neural network, which is additionally available on a mobile application and not tied to specific dermatoscopic hardware.

The main weaknesses of the study were the assessment of raters in artificial settings with no access to patient medical records or clinical features, the use of retrospectively collected test data, and a disproportional sampling design without external validation. Only seven raters participated in the study, as it was single-center. Furthermore, the test dataset size was small in comparison to the training dataset, as human raters are unable to classify large numbers of dermatoscopic images. Our test dataset size (*n* = 100) was the same size as that used in studies by Haenssle et al. [21,22,23,24,25]. The relatively small sample size led to wide confidence intervals, which overlapped for most of the results.

An additional weakness was that only three types of skin lesions were used in the test dataset, and certain melanoma subtypes, such as acral lentiginous or amelanotic melanomas, were not included. Lesions were mostly located on the trunk and extremities. Lesions on the face, scalp, and palmoplantar surfaces were not included in the test dataset. Therefore, only the most common types and locations of melanomas were addressed in the study. Lentigo maligna melanoma possesses a pigmented pseudonetwork and light brown structureless areas, which indicates malignant melanocyte distribution in follicular units [39]. Such dermatoscopic features were absent in the current test dataset. In addition, certain age groups, such as adolescents, can present with certain melanoma subtypes more frequently. Therefore, the inclusion of more variants, such as Spitzoid melanomas [40], would be beneficial for a more inclusive NNM classification performance assessment. Unfortunately, the HAM10000, MSK-1/5, and UDA2 databases that were used to form the test dataset did not include information about the reasons for benign lesion removal, nor about seborrheic keratosis subtypes. While the dermatoscopic features of most benign skin lesions have been described [41], some can still present as melanoma mimickers [42]. The performance on a test dataset of dermatoscopically equivocal lesions may herald lower classification accuracies for both the NNM and dermatologists.

The summarized sensitivities and specificities of smartphone-based artificial intelligence algorithms are presented in Table 6. Our NNM achieved a higher melanoma classification performance than that of the dermatoscopic image-based CNN from the study by Veronese et al. [26]. The study assessed a similar number (*n* = 97) of dermatoscopic images from the ISIC archive to evaluate the accuracy of a CNN algorithm, trained on 600 images. Thus, we believe that our multiclass-classification NNM would be a better aid for beginners in dermatoscopy and family physicians, as it also has a wider hardware compatibility. Sangers et al. [28] prospectively validated a previously tested smartphone-based CNN that was trained on clinical images [27] for the classification of 18 different skin tumor types. The algorithm achieved a sensitivity and specificity of 81.8% and 73.3% for melanocytic skin lesions, respectively, as well as an overall 86.9% sensitivity and 70.4% specificity for all skin tumors. Although the range of included skin lesions was greater than that in our study, only clinical images were used. Therefore, it is difficult to directly compare its accuracy with that of our classification model, which used dermatoscopic images.

While the adoption rate of dermatoscopy amongst primary care physicians continues to be limited, the expanding domains of artificial intelligence and teledermatology warrant further research, with the aim of introducing cutting-edge technical assistance [4]. The remarkable potential of contemporary smartphone cameras, combined with breakthroughs in machine learning, enables the seamless integration of these technologies into handheld systems, optimized for use in primary care environments. Such accessibility would equip family practitioners with the capability of performing the dermatologist-equivalent categorization of benign and malignant skin lesions. Consequently, this would considerably enhance patient accessibility [43], in addition to curtailing healthcare costs [44]. This is particularly important in rural areas where the access to dermatologists may be limited.

The primary goal of this study was the demonstration of the real-life application possibilities of state-of-the-art deep learning algorithms implemented in common devices. Our results show that the proposed solution can give very promising results and lead to the development of self-monitoring technologies. As we already demonstrated, the proposed algorithm can be used as a mobile application—individual users can perform self-examinations of skin lesions using any image acquisition device. This can help raise the awareness of skin cancer and encourage early detection. The proposed solution can be employed in public health campaigns to screen large populations and identify high-risk individuals who may require a more detailed examination.

Our study assessed the accuracy of an NNM in classifying melanomas, melanocytic nevi, and seborrheic keratoses. However, the algorithm should be improved in order to increase the sensitivity for seborrheic keratoses and to include more non-melanocytic skin lesions and different melanoma subtypes, including those in difficult-to-assess locations, such as the face and palmoplantar surfaces. Additionally, further validation of the smartphone-based YOLO network is needed through the use of external datasets, a greater number of dermatoscopic images, and, ultimately, prospective multicenter studies. Future research should assess whether the classification performance of family physicians improves when using smartphone-based artificial intelligence algorithms in a prospective study, in order to display the clear benefit of such technologies in daily clinical practice.

## Figures and Tables

**Table 1:** Characteristics of the test dataset.

Characteristics	Number	Percent
**Patients**	**100**	
Mean age (SD), years	55.4 (±15.8)	
Sex		
Male	54	54
Female	46	46
**Assessed lesions**	**100**	
Image datasets *		
HAM 10000	38	38
MSK-1	23	23
MSK-2	14	14
MSK-3	3	3
MSK-4	16	16
MSK-5	5	5
UDA2	1	1
Lesion classes		
Melanoma	32	32
Melanocytic nevus	35	35
Seborrheic keratosis	33	33
Localization		
Head and neck	4	4
Upper extremities	19	19
Lower extremities	20	20
Anterior torso	16	16
Lateral torso	2	2
Posterior torso	26	26
Not specified	6	6

SD—standard deviation. *—data sources for the test dataset.

**Table 2:** The effectiveness of the neural network model in comparison with the classification performance of dermatologists.

Rater Level	Melanoma		Melanocytic Nevus		Seborrheic Keratosis	
	Sensitivity	Specificity	Sensitivity	Specificity	Sensitivity	Specificity
Skilled	0.98 (0.92–1.00)	0.84 (0.51–0.96)	0.73 (0.33–0.94)	0.98 (0.88–1.00)	0.89 (0.58–0.98)	0.99 (0.92–1.00)
Beginners	0.83 (0.77–0.87)	0.85 (0.77–0.90)	0.66 (0.57–0.74)	0.87 (0.80–0.92)	0.73 (0.52–0.87)	0.89 (0.83–0.93)
All raters	0.87 (0.79–0.92)	0.84 (0.76–0.90)	0.68 (0.56–0.78)	0.90 (0.83–0.95)	0.78 (0.60–0.89)	0.91 (0.85–0.95)
NNM	0.88 (0.71–0.96)	0.87 (0.76–0.94)	0.77 (0.60–0.90)	0.91 (0.81–0.97)	0.52 (0.34–0.69)	0.93 (0.83–0.98)

Data are presented with a 95% confidence interval given in the parentheses.

**Table 3:** The estimated difference between the classification performance of the neural network model and human raters.

Rater Level	Melanoma		Melanocytic Nevus		Seborrheic Keratosis	
	Sensitivity	Specificity	Sensitivity	Specificity	Sensitivity	Specificity
Skilled	−0.11 (−0.27, 0.00)	0.03 (−0.13, 0.37)	0.04 (−0.23, 0.46)	−0.07 (−0.17, 0.04)	−0.38 (−0.58, −0.02)	−0.06 (−0.15, 0.02)
Beginners	0.05 (−0.12, 0.15)	0.02 (−0.10, 0.13)	0.11 (−0.08, 0.26)	0.03 (−0.08, 0.13)	−0.22 (−0.45, 0.06)	0.04 (−0.06, 0.12)
All raters	0.00 (−0.17, 0.12)	0.02 (−0.10, 0.13)	0.09 (−0.11, 0.26)	0.00 (−0.10, 0.10)	−0.26 (−0.48, −0.01)	0.01 (−0.09, 0.09)

Data are presented with a 95% confidence interval given in the parentheses.

**Table 4:** Sensitivity and specificity of each individual human rater.

Rater Level	Melanoma		Melanocytic Nevus		Seborrheic Keratosis	
	Sensitivity	Specificity	Sensitivity	Specificity	Sensitivity	Specificity
Beginner 1	0.91 (0.75–0.98)	0.93 (0.84–0.98)	0.57 (0.39–0.74)	0.97 (0.89–1.00)	0.97 (0.84–1.00)	0.82 (0.71–0.90)
Beginner 2	0.84 (0.67–0.95)	0.84 (0.73–0.92)	0.80 (0.63–0.92)	0.82 (0.70–0.90)	0.58 (0.39–0.75)	0.96 (0.87–0.99)
Beginner 3	0.78 (0.60–0.91)	0.90 (0.80–0.96)	0.60 (0.42–0.76)	0.92 (0.83–0.97)	0.97 (0.84–1.00)	0.85 (0.74–0.93)
Beginner 4	0.78 (0.60–0.91)	0.85 (0.75–0.93)	0.74 (0.57–0.88)	0.86 (0.75–0.93)	0.64 (0.45–0.80)	0.87 (0.76–0.94)
Beginner 5	0.81 (0.64–0.93)	0.72 (0.60–0.82)	0.60 (0.42–0.76)	0.80 (0.68–0.89)	0.52 (0.34–0.69)	0.94 (0.85–0.98)
Skilled 1	0.97 (0.84–1.00)	0.71 (0.58–0.81)	0.51 (0.34–0.69)	0.95 (0.87–0.99)	0.79 (0.61–0.91)	0.97 (0.90–1.00)
Skilled 2	1.00 (0.89–0.96)	0.97 (0.90–1.00)	0.94 (0.81–0.99)	1.00 (0.94–1.00)	1.00 (0.89–1.00)	1.00 (0.95–1.00)

Data are presented with a 95% confidence interval given in the parentheses.

**Table 5:** The inter-observer agreement using the Fleiss kappa for different rater groups and skin lesion classes.

Rater Level	Fleiss Kappa			
	Melanoma	Melanocytic Nevus	Seborrheic Keratosis	All Lesion Classes
Skilled	0.57 (0.37–0.77)	0.49 (0.30–0.69)	0.79 (0.59–0.98)	0.62 (0.48–0.76)
Beginners	0.56 (0.50–0.62)	0.43 (0.37–0.50)	0.50 (0.44–0.56)	0.50 (0.46–0.54)
All raters	0.56 (0.51–0.60)	0.46 (0.42–0.50)	0.56 (0.52–0.61)	0.53 (0.50–0.56)

Data are presented with a 95% confidence interval given in the parentheses.

**Table 6:** Sensitivity and specificity of the neural network model and other studies employing smartphone-based artificial intelligence algorithms for dermatoscopic images.

Study	Melanoma		Melanocytic Nevus		Seborrheic Keratosis	
	Sensitivity	Specificity	Sensitivity	Specificity	Sensitivity	Specificity
NNM	0.88 (0.71–0.96)	0.87 (0.76–0.94)	0.77 (0.60–0.90)	0.91 (0.81–0.97)	0.52 (0.34–0.69)	0.93 (0.83–0.98)
Veronese et al. [18]	0.84	0.82	N/A	N/A	N/A	N/A
Udrea et al. [19] *	0.93 (0.88–0.96)	N/A	N/A	0.78 (0.77–0.79)	N/A	0.78 (0.77–0.79)
Sangers et al. [20] *	0.82 (0.59–0.95)	0.73 (0.66, 0.80)	N/A	0.80 (0.76–0.84)	N/A	0.80 (0.76–0.84)

Where available, data are presented with a 95% confidence interval given in the parentheses. NNM—neural network model. * Non-dermatoscopic images. N/A—data not available.

## Data Availability

The data presented in this study are available from the corresponding author upon request. The data are not publicly available due to privacy restrictions. Additionally, publicly available datasets were analyzed in this study. These data can be found at the following address: https://www.isic-archive.com accessed on 29 May 2023.
